# Supplementary material for: Conformational dynamics in crystals reveal the molecular bases for D76N beta-2 microglobulin aggregation propensity
Source: Nat Commun. 2018 Apr 25;9:1658. doi: 10.1038/s41467-018-04078-y (PMC5916882; doi:10.1038/s41467-018-04078-y)
Supplement: Supplementary file 1 — Supplementary Information [file 41467_2018_4078_MOESM1_ESM.pdf]

Conformational dynamics in crystals reveal the molecular bases for D76N Beta-2 microglobulin aggregation propensity

Le Marchand et al.

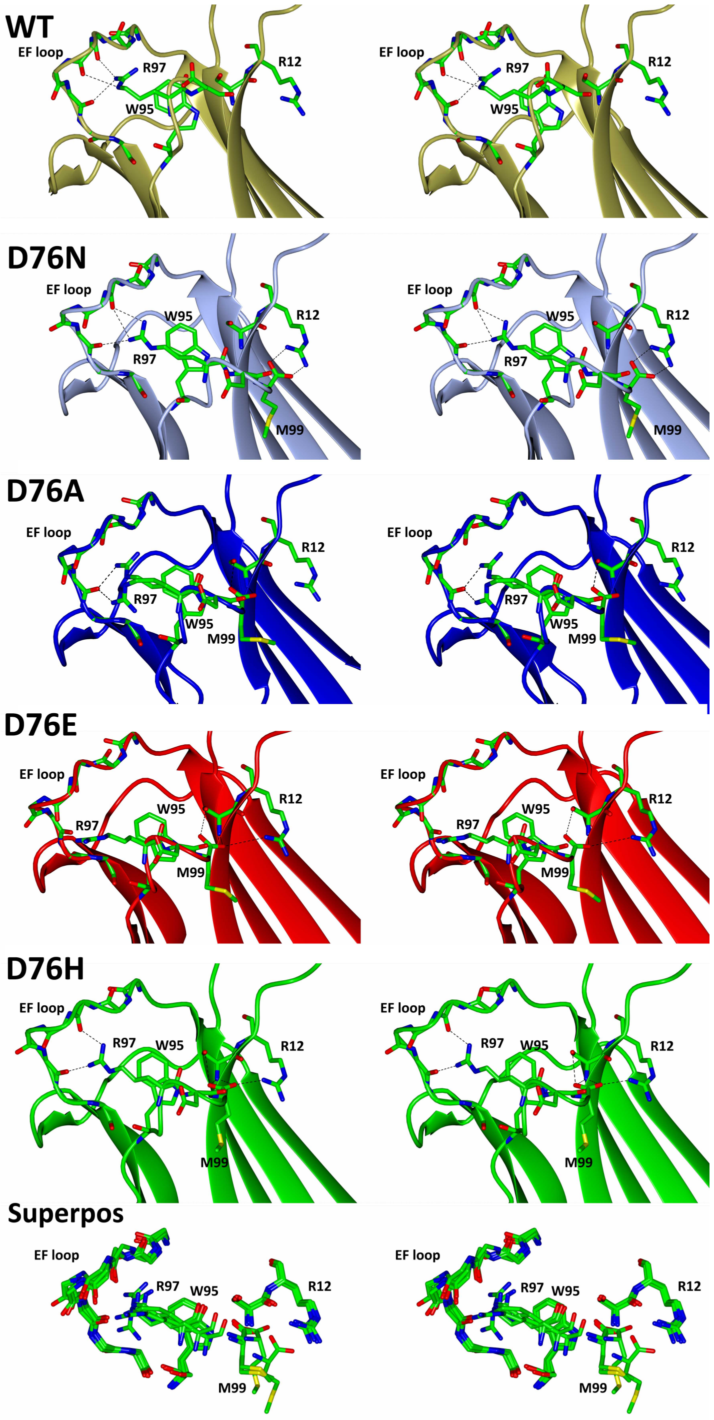

Supplementary Figure 1: Stereo view of the apical region of wt β2m (gold), D76N (light blue), D76A (blue), D76E (red), D76H (green) (2YXF, 4FXL, 4RMW, 4RMU, 4RMV, respectively). H-bonds between Arg97 and the EF loop or Met99 and the residues 11-12 are shown by dashed lines. In the structure of wt β2m Met99 is not present. In the sixth panel: Superposition of all the C-*termini* from the above structures highlighting that while the regions interacting with Arg97 and Met99 (EF loop main chain, Trp95 and the residues 11-12) are well superposable, C-*termini* display a notable structural variability.

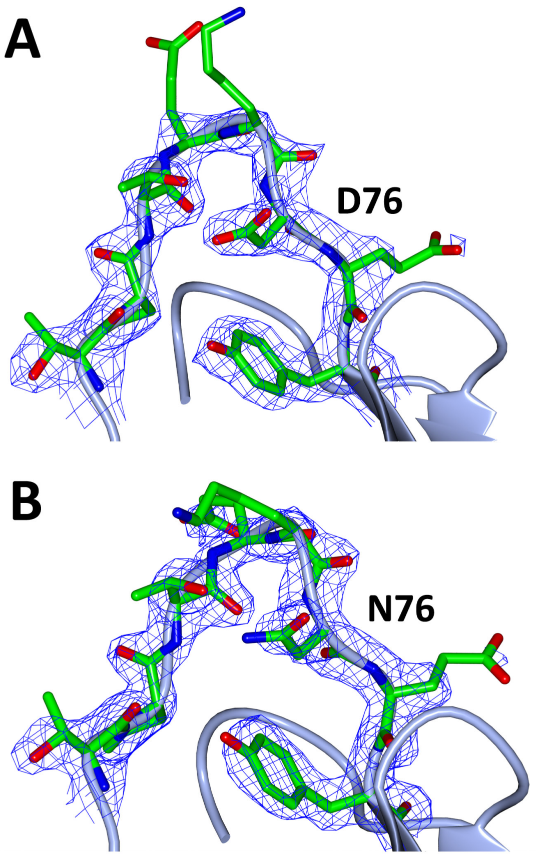

Supplementary Figure 2: A-B) Representation as cylinder of the EF loop of wt β2m (A) and of D76N (B) from data collected at room temperature. Electron density (contoured at 1.2 σ) is clipped around the loop.

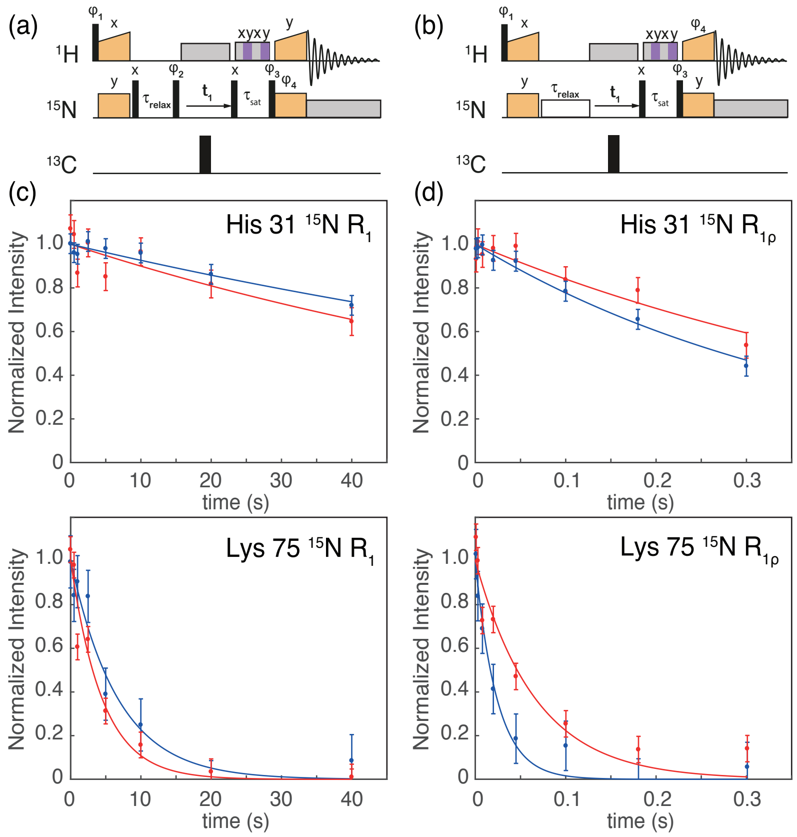

Supplementary Figure 3: a), b) Pulse sequences used for ^{15}N R_1 (a) and ^{15}N $R_{1\rho}$ (b) relaxation rate measurement. Narrow and wide rectangles indicate respectively 90° and 180° pulses. Cross-Polarization (CP) transfers between ^{1}H and ^{15}N (orange boxes) were performed with a linearly ramped pulse from 90% to 100% of a maximum frequency 100 kHz on ^{1}H and a rectangular pulse on ^{15}N of 40 kHz. CP lengths were set to respectively 900 and 600 µs for ^{1}H to ^{15}N and ^{15}N to ^{1}H transfers. Water suppression was achieved by storing ^{15}N magnetization along the *z* axis and applying the MISSISSIPI pulse sequence with a 19 kHz irradiation during 300 ms. Grey rectangles indicate heteronuclear decoupling using WALTZ-16 with 10 kHz rf power. The phase cycles were: φ1=[y, -y], φ2=[8(-x), 8(x)], φ3=[2(-x), 2(x)], φ4=[4(y), 4(-y)]. The receiver phase cycles were [y, −y, −y, y, −y, y, y, −y, −y, y, y, −y, y, −y, −y, y] for a) and [y, −y, −y, y, −y, y, y, −y] for b). c) d) Examples of relaxation decay curves for wt (red) and D76N (blue) β2m: (c) ^{15}N R_1 (d) ^{15}N $R_{1\rho}$ at 20 kHz spin-lock field. Error bars (s.d.) was evaluated from 1000 Monte-Carlo simulations.

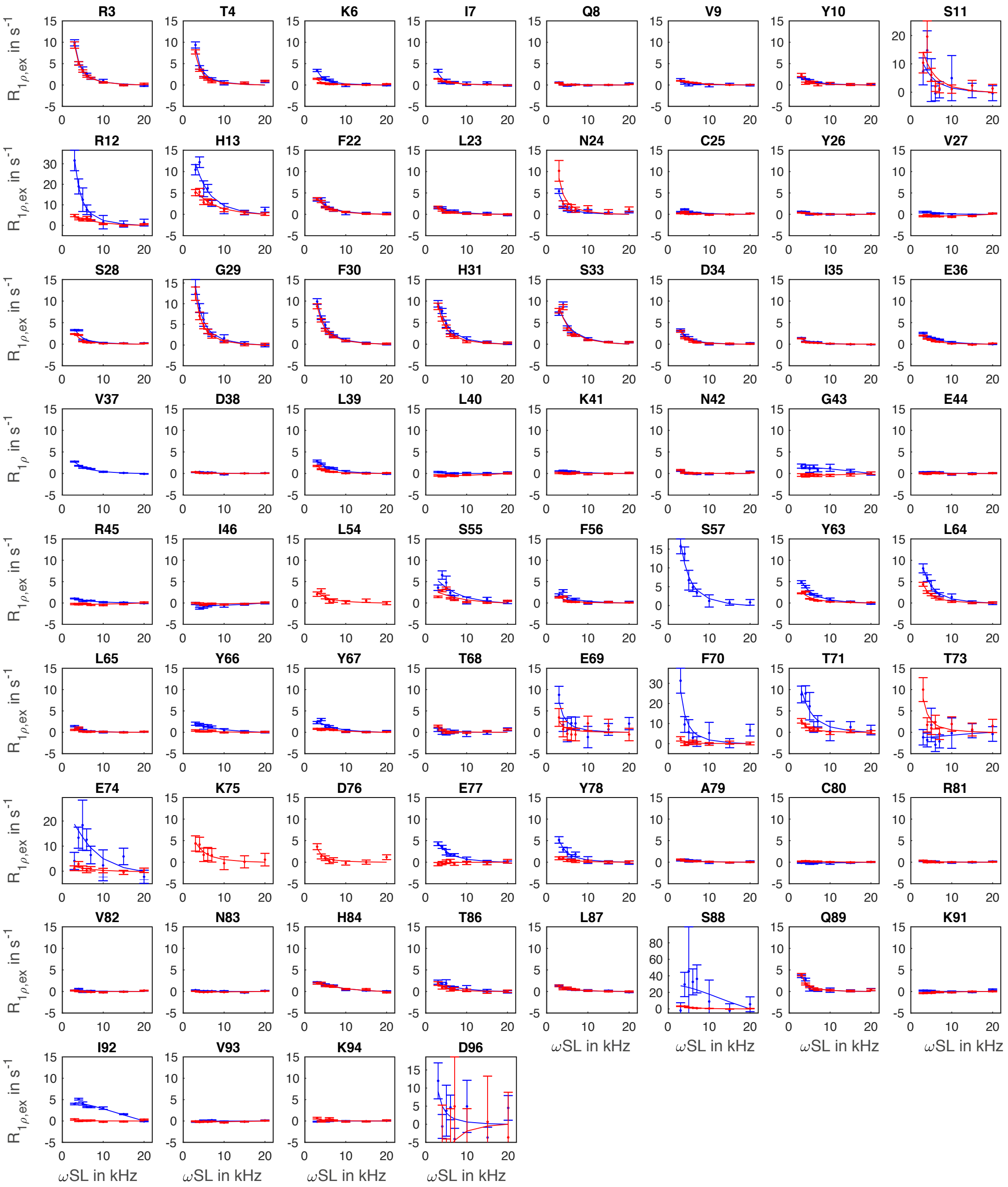

Supplementary Figure 4: ^{15}N $R_{1\rho}$ relaxation dispersion curves for D76N (blue) and wt (red) β2m acquired on a 1 GHz spectrometer. Best fit Lorentzian functions are plotted with solid lines. Error bars (s.d.) was evaluated from 1000 Monte-Carlo simulations.

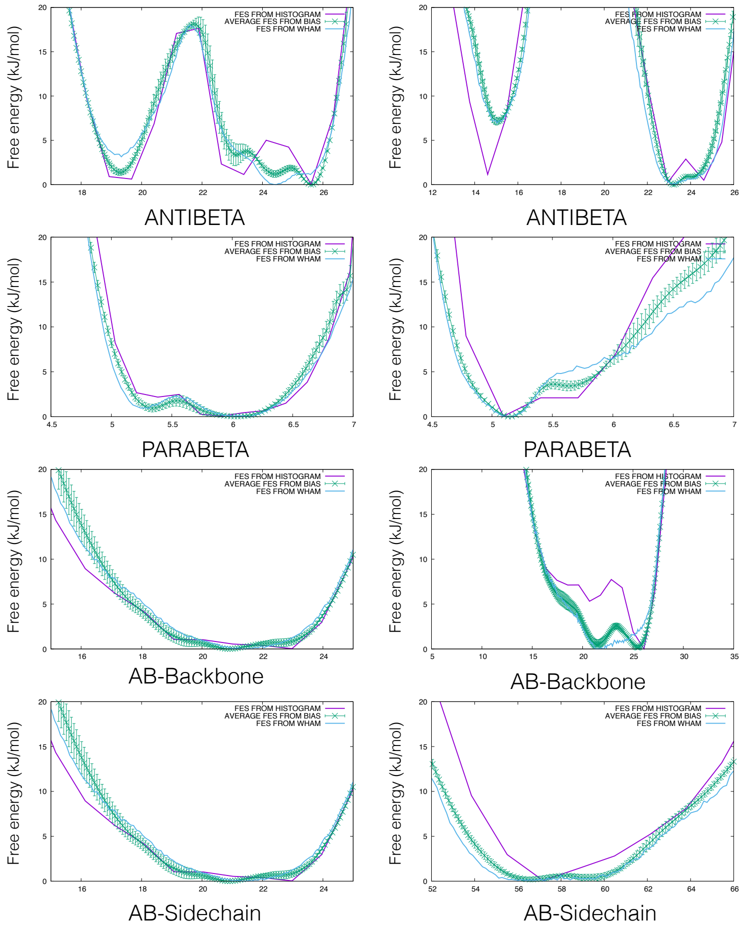

Supplementary Figure 5: Convergence of the Replica-Averaged Metadynamics simulations, wt β2m (left) and D76N (right). The plots show the comparison between the free energy obtained by taking the histogram from the second half of the simulation and rescaling it for the Metadynamics bias factor; the free energy obtained from the metadynamics bias averaged over the last 50 ns of simulation; the free energy obtained from a weighted histogram analysis. The maximum deviation between free energies is always less than 5 kJ/mol and on average less than 2 kJ/mol.

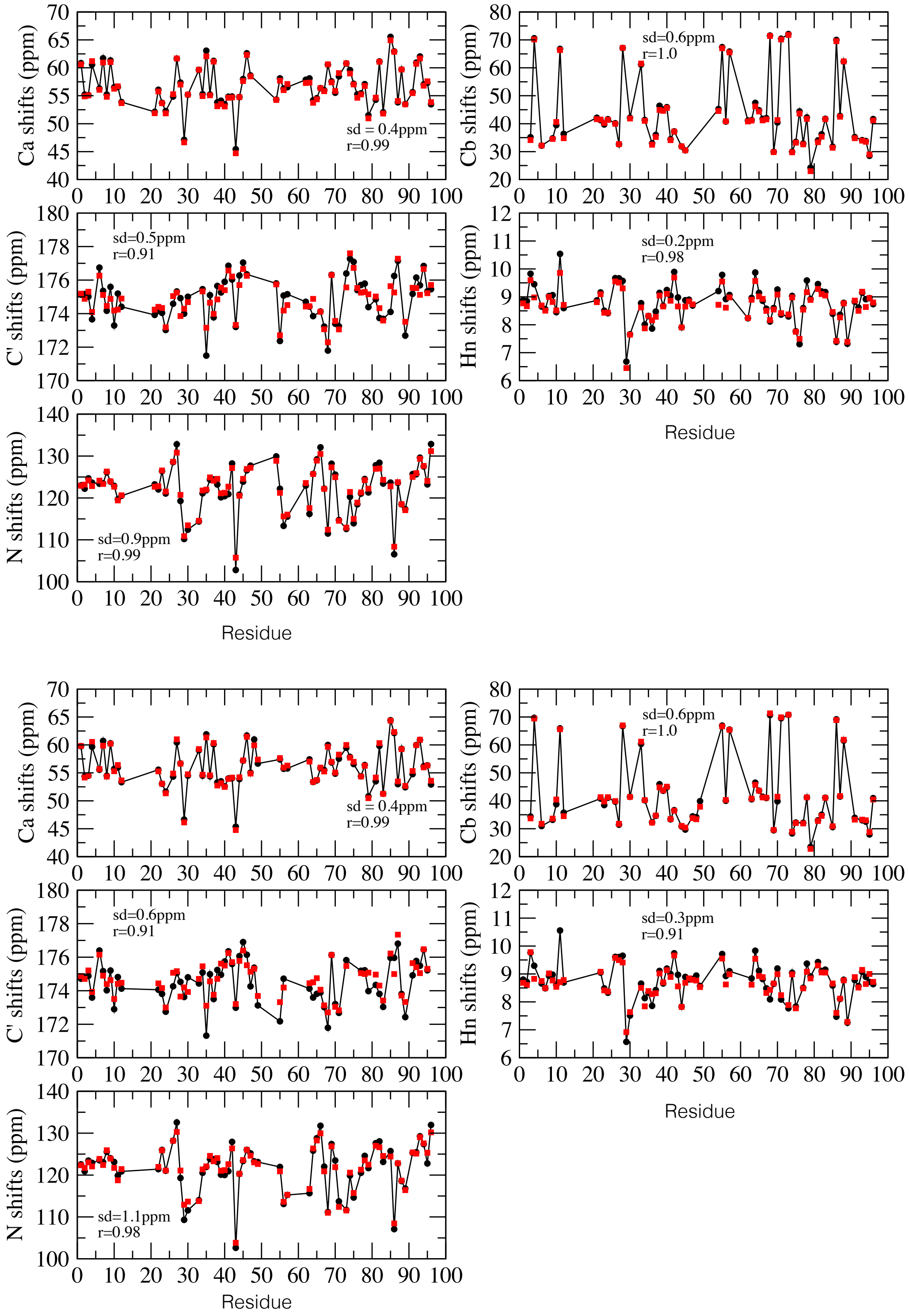

Supplementary Figure 6: Validation of the wt β2m ensemble (top) and D76N ensemble (bottom) by comparing the calculated chemical shifts (red) with the experimental chemical shifts used as restraints (black). Average deviations and correlations are reported.

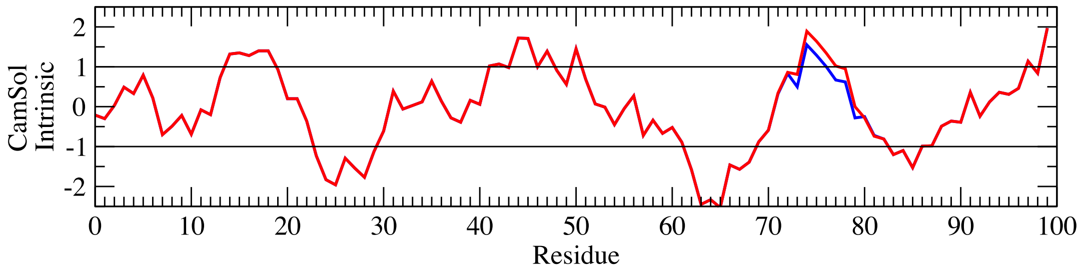

Supplementary Figure 7: Sequence solubility profile calculated using CamSol. The black curve is for the wt sequence while the red curve is of the D76N mutant. Differences in the profiles are localized around position 76. Values larger than 1 are related to sites strongly contributing to the solubility, while values lesser then -1 indicate aggregation prone sites.

Supplementary Table 1: Crystal structures: data collection and refinement statistics.

	**D76E**	**D76A**	**D76H**	**R97Q**	**wt RT**	**D76N RT**
**PDB ID**	4RMU	4RMW	4RMV	5CSG	5CS7	5CSB
**Wavelength (Å)**	0.9840	0.8710	0.9840	0.9840	0.9840	0.9840
**Resolution range (Å)**	47.9 - 1.40 (1.45 - 1.40)	30.9 - 1.40 (1.45 - 1.40)	20.4 - 1.46 (1.52 - 1.46)	38.8 - 1.50 (1.55 - 1.50)	39.0 - 2.10 (2.18 - 2.10)	45.0 - 1.72 (1.78 - 1.72)
**Space group**	C 1 2 1	C 1 2 1	C 1 2 1	I 1 2 1	I 1 2 1	I 1 2 1
**Unit cell (Å)**	77.40 Å; 28.87 Å; 57.87Å; β=124.17°	77.62 Å; 28.99 Å; 56.29 Å; β=127.20°	77.54 Å; 28.87 Å; 53.28 Å; β=125.78°	57.35 Å; 29.07 Å; 61.24 Å; β=98.06°	59.63 Å; 29.24 Å; 65.96 Å; β=102.89 °	56.00 Å; 29.32 Å; 63.68 Å; β=98.57°
**Unique reflections**	20395 (1615)	19920 (1982)	16363 (1650)	15172 (1462)	6598 (631)	10186 (1007)
**Multiplicity**	4.2	3.9	4.0	2.5	3.1	2.2
**Completeness (%)**	96	100	97	93	99	91
**Wilson B-factor ($Å^2$)**	11.49	11.77	15.09	16.98	30.07	23.16
**R-merge [a]**	0.050	0.066	0.075	0.065	0.079	0.056
**Reflections used in refinement**	20394	19919	16321	15146	6598	10182
**R-work [b]**	0.163 (0.165)	0.180 (0.263)	0.187 (0.306)	0.181 (0.315)	0.174 (0.242)	0.183 (0.256)
**R-free [b]**	0.192 (0.201)	0.213 (0.296)	0.229 (0.359)	0.225 (0.386)	0.238 (0.326)	0.223 (0.308)
**RMS (bonds)**	0.015	0.020	0.007	0.015	0.009	0.009
**RMS (angles °)**	1.61	2.01	1.30	1.75	1.43	1.61
**Ramachandran favoured (%)**	97	92	99	96	97	95
**Ramachandran allowed (%)**	3	7	1	4	3	5
**Rotamer outliers (%)**	1	5.5	1.9	3.5	1	0.93
**Average B-factor ($Å^2$)**	18.60	23.16	28.21	27.78	43.52	38.81
**polypeptide**	16.26	21.77	27.26	26.67	43.38	38.50
**ligands**	42.03	44.75	45.10	49.76		
**solvent**	30.59	32.01	36.57	36.87	46.78	46.25

(previous page)

[a] $R_{merge} = \Sigma_{hkl} \Sigma_j |I_{hkl,j} - \langle I_{hkl}\rangle| / \Sigma_{hkl} \Sigma_j I_{hkl,j}$, where I_{hkl} is the observed intensity and $\langle I_{hkl}\rangle$ is the average intensity for the hkl reflection.

[b] $R_{work} = \Sigma_{hkl}|F_o - F_c|/\Sigma_{hkl}F_o$ for all data except 5–10%, which were used for the R_{free} calculation.

Values given in parenthesis refer to the high-resolution shell.

Supplementary Table 2: R.m.s.d values (Å) calculated over 99 Cα atoms for the crystal structures of the following variants: D76E, D76H, D76A, R97Q, wt and D76N (collected at room temperature; RT) superimposed to the wt or D76N structures.

	D76N	D76E	D76A	D76H	R97Q	wt RT	D76N RT
wt β2m	0.59	0.16	0.61	0.39	0.20	0.31	0.40
D76N	-	0.67	0.88	0.43	0.72	0.72	0.70

Supplementary Table 3: Forward primers for the mutagenesis of the β2m sequence.

β2m mutant	Forward primers
D76N	`caccccactgaaaaaaatgagtatgcctgcc`
D76A	`caccccactgaaaaagcagagtatgcctgcc`
D76E	`caccccactgaaaaagaagagtatgcctgcc`
D76H	`caccccactgaaaaacatgagtatgcctgcc`
D76K	`caccccactgaaaaaaaagagtatgcctgcc`
R97Q	`gatagttaagtgggatcaagacatgtaaaagc`
