# Supplementary material for: Conformational dynamics in crystals reveal the molecular bases for D76N beta-2 microglobulin aggregation propensity
Source: Nat Commun. 2018 Apr 25;9:1658. doi: 10.1038/s41467-018-04078-y (PMC5916882; doi:10.1038/s41467-018-04078-y)
Supplement: Supplementary file 3 — Description of Additional Supplementary Files [file 41467_2018_4078_MOESM3_ESM.pdf]

Description of Additional Supplementary Files

File Name: Supplementary Dataset 1
Description: This contains 1H, 15N and 13C chemical shifts in ppm for D76N β2m.

File Name: Supplementary Dataset 2
Description: This contains 15N R1 and 15N R1ρ relaxation rates obtained for wt and D76N β2m.

File Name: Supplementary Dataset 3
Description: This contains model free analysis of the 15N relaxation rates for wt and D76N β2m.
