# Supplementary material for: Conformational dynamics in crystals reveal the molecular bases for D76N beta-2 microglobulin aggregation propensity
Source: Nat Commun. 2018 Apr 25;9:1658. doi: 10.1038/s41467-018-04078-y (PMC5916882; doi:10.1038/s41467-018-04078-y)
Supplement: Supplementary file 4 — Supplementary Data 1 [file 41467_2018_4078_MOESM4_ESM.docx]

**Supplementary Dataset 1:** ^1^H, ^15^N and ^13^C chemical shifts in ppm for D76N β2m, referenced to DSS, without isotope correction.

| Residue | δ(^1^H^N^) | δ(^15^N) | δ(^13^CA) | δ(^13^CB) | δ(^13^C’) |
| --- | --- | --- | --- | --- | --- |
| M0 |  |  |  |  | 171.35 |
| I1 | 8.80 | 122.56 | 59.77 | 41.66 | 174.72 |
| Q2 | 8.71 | 120.90 | 54.39 | 29.95 | 174.78 |
| R3 | 9.76 | 123.45 | 54.50 | 34.35 | 174.89 |
| T4 | 9.29 | 122.92 | 59.66 | 69.71 | 173.59 |
| P5 |  |  | 62.03 | 31.41 | 176.18 |
| K6 | 8.66 | 123.54 | 55.56 | 30.95 | 176.40 |
| I7 | 8.49 | 123.07 | 60.72 | 40.99 | 175.17 |
| Q8 | 8.94 | 125.53 | 54.50 | 33.24 | 174.03 |
| V9 | 8.98 | 124.02 | 60.28 | 33.19 | 175.21 |
| Y10 | 8.60 | 123.11 | 55.71 | 38.77 | 172.90 |
| S11 | 10.56 | 119.88 | 55.96 | 65.94 | 174.82 |
| R12 | 8.70 | 120.87 | 53.33 | 35.74 | 174.13 |
| H13 | 8.83 | 119.34 | 51.02 | 28.16 | 172.68 |
| P14 |  |  |  |  |  |
| A15 |  |  |  |  |  |
| E16 |  |  |  |  |  |
| N17 |  |  |  |  |  |
| G18 |  |  |  |  |  |
| K19 |  |  |  |  |  |
| S20 |  |  |  |  |  |
| N21 |  |  | 51.58 | 44.08 | 173.88 |
| F22 | 9.05 | 121.40 | 55.56 | 40.81 | 174.06 |
| L23 | 8.48 | 126.00 | 53.04 | 38.46 | 173.81 |
| N24 | 8.33 | 121.03 | 51.64 | 41.08 | 172.75 |
| C25 | 9.47 | 121.18 | 53.77 | 41.26 | 171.72 |
| Y26 | 9.61 | 128.19 | 54.33 | 39.68 | 174.26 |
| V27 | 9.61 | 132.56 | 60.44 | 31.46 | 175.05 |
| S28 | 9.65 | 119.29 | 56.69 | 66.75 | 174.53 |
| G29 | 6.57 | 109.30 | 46.65 |  | 173.62 |
| F30 | 7.51 | 111.58 | 54.50 | 41.44 | 174.80 |
| H31 | 8.55 | 117.64 | 57.47 |  | 174.75 |
| P32 |  |  | 62.26 | 34.80 | 176.10 |
| S33 | 8.65 | 114.01 | 59.04 | 60.37 | 174.43 |
| D34 | 8.14 | 120.54 | 54.50 | 40.18 | 175.09 |
| I35 | 8.32 | 121.93 | 61.90 | 39.02 | 171.32 |
| E36 | 7.86 | 123.84 | 54.39 | 32.29 | 174.99 |
| V37 | 8.42 | 123.77 | 60.11 | 34.62 | 173.51 |
| D38 | 9.11 | 123.12 | 53.29 | 45.93 | 175.24 |
| L39 | 8.66 | 120.09 | 53.49 | 43.59 | 174.95 |
| L40 | 9.19 | 120.01 | 52.59 | 44.85 | 175.74 |
| K41 | 9.00 | 120.90 | 53.99 | 33.36 | 176.35 |
| N42 | 9.74 | 127.91 | 54.05 | 36.64 | 175.59 |
| G43 | 8.97 | 102.62 | 45.36 |  | 172.98 |
| E44 | 7.83 | 120.28 | 53.88 | 30.85 | 176.06 |
| R45 | 8.90 | 123.35 | 57.19 | 29.69 | 176.90 |
| I46 | 8.81 | 126.02 | 61.68 | 37.30 | 176.15 |
| E47 |  |  |  |  |  |
| K48 |  |  |  |  |  |
| V49 |  |  |  |  |  |
| E50 |  |  |  |  |  |
| H51 |  |  |  |  |  |
| S52 |  |  |  |  |  |
| D53 |  |  |  |  |  |
| L54 |  |  | 53.55 | 44.08 | 175.36 |
| S55 | 9.72 | 121.94 | 57.41 | 66.93 | 172.18 |
| F56 | 8.93 | 113.09 | 55.73 | 40.00 | 174.72 |
| S57 | 9.10 | 115.25 | 55.79 | 65.49 |  |
| K58 |  |  |  |  |  |
| D59 |  |  |  |  |  |
| W60 |  |  |  |  |  |
| S61 |  |  |  |  |  |
| F62 |  |  | 57.41 | 40.42 | 174.41 |
| Y63 | 8.84 | 115.66 | 57.41 | 40.54 | 174.13 |
| L64 | 9.83 | 125.83 | 53.38 | 46.48 | 173.59 |
| L65 | 9.11 | 128.79 | 53.66 | 43.64 | 173.81 |
| Y66 | 8.82 | 131.77 | 55.84 | 41.46 | 173.89 |
| Y67 | 8.49 | 122.01 | 55.51 | 40.99 | 173.02 |
| T68 | 8.09 | 111.17 | 60.00 | 70.69 | 171.79 |
| E69 | 8.65 | 127.40 | 56.85 | 29.43 | 176.13 |
| F70 | 9.19 | 123.46 | 54.85 | 39.82 | 173.20 |
| T71 | 8.08 | 113.71 | 57.53 | 69.53 | 172.68 |
| P72 |  |  | 62.26 | 31.50 | 176.72 |
| T73 | 7.77 | 111.67 | 59.49 | 70.78 | 175.83 |
| E74 | 9.04 | 119.88 | 57.86 | 28.34 |  |
| K75 | 7.83 | 114.64 | 56.57 | 32.20 | 175.78 |
| N76 | 7.25 | 114.28 | 51.97 | 41.56 | 173.27 |
| E77 | 8.51 | 120.54 | 54.39 | 32.11 | 175.19 |
| Y78 | 9.37 | 124.58 | 56.41 | 41.17 | 175.22 |
| A79 | 8.91 | 121.66 | 50.80 | 23.49 | 173.98 |
| C80 | 9.20 | 119.37 | 52.65 | 43.68 | 171.64 |
| R81 | 9.43 | 127.61 | 53.49 | 32.98 | 174.35 |
| V82 | 9.11 | 128.03 | 59.83 | 35.00 | 173.81 |
| N83 | 9.16 | 123.14 | 51.28 | 41.08 | 173.03 |
| H84 | 7.99 | 122.94 | 56.80 | 35.25 | 175.49 |
| V85 | 8.59 | 125.72 | 64.43 | 30.61 | 174.02 |
| T86 | 7.47 | 107.08 | 62.13 | 69.17 | 175.96 |
| L87 | 8.10 | 122.75 | 52.98 | 41.53 | 176.81 |
| S88 | 8.79 | 118.57 | 59.30 | 61.54 | 173.81 |
| Q89 | 7.25 | 116.78 | 52.42 | 29.14 | 172.43 |
| P90 |  |  | 63.38 | 30.64 | 176.16 |
| K91 | 8.78 | 125.33 | 54.78 | 33.90 | 174.92 |
| I92 | 8.57 | 125.45 | 59.97 | 37.76 | 175.78 |
| V93 | 9.08 | 129.25 | 60.89 | 33.03 | 175.49 |
| K94 | 8.89 | 127.37 | 56.18 | 32.53 | 176.45 |
| W95 | 8.72 | 122.77 | 56.35 | 27.96 | 175.22 |
| D96 | 8.64 | 131.96 | 52.93 | 40.95 |  |
| R97 |  |  |  |  |  |
| D98 |  |  |  |  |  |
| M99 |  |  |  |  |  |
