# Supplementary material for: Conformational dynamics in crystals reveal the molecular bases for D76N beta-2 microglobulin aggregation propensity
Source: Nat Commun. 2018 Apr 25;9:1658. doi: 10.1038/s41467-018-04078-y (PMC5916882; doi:10.1038/s41467-018-04078-y)
Supplement: Supplementary file 5 — Supplementary Data 2 [file 41467_2018_4078_MOESM5_ESM.docx]

**Supplementary Dataset 2:** ^15^N R_1_ and ^15^N R_1ρ_ relaxation rates obtained for wt and D76N β2m on an 18.8 T spectrometer (in ms^-1^ and s^-1^, respectively).

| Residue number | WT | | | | D76N | | | |
| --- | --- | --- | --- | --- | --- | --- | --- | --- |
|  | ^15^N R_1_ ± error | | ^15^N R_1ρ_ ± error | | ^15^N R_1_ ± error | | ^15^N R_1ρ_ ± error | |
| 1 | 66,35 | 2,77 | 5,26 | 0,19 | 49,22 | 1,07 | 8,04 | 0,18 |
| 2 | 42,42 | 1,07 | 6,39 | 0,14 |  |  |  |  |
| 3 | 24,71 | 1,23 | 1,73 | 0,10 | 20,85 | 0,67 | 1,72 | 0,07 |
| 4 | 37,28 | 0,77 | 4,52 | 0,08 | 31,07 | 0,70 | 4,58 | 0,11 |
| 5 |  |  |  |  |  |  |  |  |
| 6 | 23,34 | 0,59 | 2,72 | 0,07 | 26,54 | 1,22 | 1,34 | 0,11 |
| 7 | 41,15 | 0,88 | 2,13 | 0,05 | 45,14 | 1,07 | 1,52 | 0,06 |
| 8 | 19,93 | 0,45 | 1,24 | 0,04 | 19,43 | 0,58 | 1,12 | 0,06 |
| 9 | 25,36 | 0,33 | 2,36 | 0,03 | 21,54 | 0,75 | 2,63 | 0,10 |
| 10 | 17,60 | 0,66 | 4,89 | 0,13 | 22,01 | 1,00 | 4,85 | 0,20 |
| 11 | 39,66 | 4,22 | 6,57 | 0,58 | 46,31 | 5,01 | 12,93 | 1,49 |
| 12 | 29,58 | 2,35 | 5,38 | 0,33 | 39,98 | 1,91 | 12,25 | 0,65 |
| 13 | 36,65 | 1,20 | 5,81 | 0,18 | 32,02 | 1,29 | 7,55 | 0,30 |
| 14 |  |  |  |  |  |  |  |  |
| 15 |  |  |  |  |  |  |  |  |
| 16 |  |  |  |  |  |  |  |  |
| 17 |  |  |  |  |  |  |  |  |
| 18 |  |  |  |  |  |  |  |  |
| 19 |  |  |  |  |  |  |  |  |
| 20 |  |  |  |  |  |  |  |  |
| 21 |  |  |  |  |  |  |  |  |
| 22 | 37,77 | 1,00 | 3,48 | 0,09 | 36,25 | 0,81 | 4,63 | 0,11 |
| 23 | 44,54 | 1,16 | 3,36 | 0,09 | 19,87 | 0,45 | 4,26 | 0,08 |
| 24 | 24,70 | 2,11 | 2,76 | 0,23 | 11,99 | 0,88 | 2,72 | 0,15 |
| 25 | 11,27 | 0,40 | 1,91 | 0,05 | 9,06 | 0,36 | 1,82 | 0,05 |
| 26 | 7,64 | 0,34 | 1,64 | 0,05 | 8,67 | 0,45 | 1,68 | 0,07 |
| 27 | 12,64 | 0,54 | 1,15 | 0,06 | 11,90 | 0,55 | 0,79 | 0,07 |
| 28 | 17,86 | 0,45 | 0,89 | 0,04 | 17,17 | 0,56 | 0,94 | 0,06 |
| 29 | 21,18 | 2,33 | 2,08 | 0,22 | 21,57 | 2,05 | 3,42 | 0,25 |
| 30 | 27,32 | 1,12 | 2,84 | 0,10 | 30,80 | 0,70 | 4,01 | 0,09 |
| 31 | 10,53 | 1,18 | 1,74 | 0,15 | 7,65 | 0,76 | 2,52 | 0,14 |
| 32 |  |  |  |  |  |  |  |  |
| 33 | 21,48 | 1,02 | 2,15 | 0,10 | 19,85 | 0,79 | 2,26 | 0,10 |
| 34 | 38,40 | 1,58 | 2,34 | 0,10 | 29,99 | 1,14 | 2,61 | 0,11 |
| 35 | 21,29 | 0,46 | 0,94 | 0,03 | 17,88 | 0,31 | 1,49 | 0,03 |
| 36 | 18,01 | 0,47 | 1,56 | 0,04 | 17,92 | 0,42 | 1,21 | 0,05 |
| 37 |  |  |  |  | 20,23 | 0,37 | 1,48 | 0,04 |
| 38 | 16,82 | 0,33 | 1,17 | 0,03 | 17,54 | 0,24 | 1,93 | 0,03 |
| 39 | 20,80 | 0,59 | 2,28 | 0,06 | 24,15 | 0,50 | 3,88 | 0,08 |
| 40 | 20,80 | 1,01 | 3,79 | 0,13 | 18,97 | 0,61 | 3,81 | 0,11 |
| 41 | 17,65 | 0,95 | 1,51 | 0,10 | 21,71 | 0,67 | 2,56 | 0,09 |
| 42 | 31,28 | 0,89 | 2,98 | 0,08 | 27,75 | 0,60 | 2,35 | 0,06 |
| 43 | 33,20 | 1,23 | 6,42 | 0,21 | 30,95 | 1,22 | 7,63 | 0,25 |
| 44 | 42,06 | 1,11 | 2,52 | 0,07 | 39,00 | 0,68 | 3,75 | 0,07 |
| 45 | 78,97 | 1,53 | 4,52 | 0,08 | 47,77 | 0,53 | 4,07 | 0,04 |
| 46 | 36,07 | 1,06 | 1,86 | 0,07 | 64,90 | 1,52 | 5,84 | 0,16 |
| 47 |  |  |  |  |  |  |  |  |
| 48 |  |  |  |  |  |  |  |  |
| 49 |  |  |  |  |  |  |  |  |
| 50 |  |  |  |  |  |  |  |  |
| 51 |  |  |  |  |  |  |  |  |
| 52 |  |  |  |  |  |  |  |  |
| 53 |  |  |  |  |  |  |  |  |
| 54 | 59,01 | 1,83 | 9,54 | 0,26 |  |  |  |  |
| 55 | 20,35 | 0,71 | 3,18 | 0,10 | 21,58 | 1,18 | 4,98 | 0,25 |
| 56 | 13,85 | 0,72 | 3,25 | 0,12 | 13,60 | 0,71 | 4,50 | 0,18 |
| 57 |  |  |  |  | 30,10 | 1,72 | 9,03 | 0,48 |
| 58 |  |  |  |  |  |  |  |  |
| 59 |  |  |  |  |  |  |  |  |
| 60 |  |  |  |  |  |  |  |  |
| 61 |  |  |  |  |  |  |  |  |
| 62 |  |  |  |  |  |  |  |  |
| 63 | 7,04 | 0,33 | 1,56 | 0,04 | 7,23 | 0,56 | 2,01 | 0,08 |
| 64 | 12,16 | 0,92 | 2,47 | 0,11 | 12,41 | 0,85 | 2,86 | 0,16 |
| 65 | 17,06 | 0,81 | 2,27 | 0,10 | 29,39 | 0,61 | 3,11 | 0,07 |
| 66 | 18,67 | 0,71 | 2,10 | 0,07 | 18,67 | 0,69 | 2,64 | 0,09 |
| 67 | 18,12 | 0,48 | 3,85 | 0,07 | 18,87 | 0,38 | 7,14 | 0,13 |
| 68 | 30,03 | 0,57 | 8,15 | 0,13 | 23,46 | 0,61 | 9,64 | 0,23 |
| 69 | 96,78 | 5,60 | 17,76 | 1,00 | 119,96 | 4,68 | 20,35 | 0,87 |
| 70 | 49,23 | 3,53 | 6,03 | 0,37 | 48,63 | 4,89 | 19,76 | 1,91 |
| 71 | 71,01 | 3,12 | 5,30 | 0,22 | 80,12 | 4,83 | 10,89 | 0,75 |
| 72 |  |  |  |  |  |  |  |  |
| 73 | 144,80 | 13,53 | 15,20 | 1,19 | 367,23 | 24,96 | 20,59 | 1,55 |
| 74 | 199,14 | 8,88 | 15,80 | 0,71 | 137,39 | 10,99 | 26,16 | 2,44 |
| 75 | 222,40 | 13,40 | 15,09 | 0,93 | 149,04 | 17,85 | 41,97 | 5,65 |
| 76 | 187,60 | 5,98 | 10,49 | 0,31 | 120,65 | 30,64 | 40,50 | 15,36 |
| 77 | 73,93 | 3,92 | 3,61 | 0,21 | 47,33 | 1,36 | 7,10 | 0,20 |
| 78 | 16,98 | 0,88 | 4,53 | 0,15 | 45,41 | 1,95 | 4,95 | 0,20 |
| 79 | 15,21 | 0,56 | 2,13 | 0,07 | 15,34 | 0,64 | 2,98 | 0,11 |
| 80 | 11,88 | 0,42 | 1,81 | 0,06 | 8,99 | 0,44 | 1,85 | 0,07 |
| 81 | 12,03 | 0,59 | 1,03 | 0,06 | 11,65 | 0,40 | 1,11 | 0,05 |
| 82 | 16,75 | 0,63 | 1,66 | 0,07 | 14,59 | 0,54 | 0,99 | 0,06 |
| 83 | 18,34 | 0,32 | 2,25 | 0,03 |  |  |  |  |
| 84 | 18,62 | 0,85 | 1,91 | 0,08 | 16,25 | 0,42 | 2,52 | 0,06 |
| 85 |  |  |  |  |  |  |  |  |
| 86 | 13,63 | 1,39 | 3,08 | 0,21 | 13,07 | 1,02 | 3,27 | 0,19 |
| 87 | 23,09 | 1,14 | 1,48 | 0,08 | 17,35 | 0,63 | 4,07 | 0,11 |
| 88 | 41,88 | 1,11 | 7,13 | 0,18 | 121,62 | 11,34 | 34,45 | 4,38 |
| 89 | 98,47 | 1,50 | 8,95 | 0,13 | 88,37 | 1,88 | 10,83 | 0,25 |
| 90 |  |  |  |  |  |  |  |  |
| 91 | 28,14 | 0,54 | 1,11 | 0,04 | 43,57 | 1,39 | 2,45 | 0,12 |
| 92 | 53,67 | 1,04 | 3,80 | 0,06 | 38,15 | 0,56 | 2,76 | 0,05 |
| 93 | 32,38 | 0,80 | 1,29 | 0,06 | 34,94 | 0,54 | 2,46 | 0,06 |
| 94 | 35,43 | 1,23 | 1,92 | 0,08 | 30,31 | 0,51 | 2,00 | 0,05 |
| 95 | 61,35 | 2,77 | 5,42 | 0,23 | 36,38 | 2,19 | 8,03 | 0,42 |
| 96 | 58,77 | 10,02 | 33,10 | 8,03 | 57,79 | 5,46 | 30,92 | 3,51 |
| 97 |  |  |  |  |  |  |  |  |
| 98 |  |  |  |  |  |  |  |  |
| 99 |  |  |  |  |  |  |  |  |
