# Supplementary material for: Conformational dynamics in crystals reveal the molecular bases for D76N beta-2 microglobulin aggregation propensity
Source: Nat Commun. 2018 Apr 25;9:1658. doi: 10.1038/s41467-018-04078-y (PMC5916882; doi:10.1038/s41467-018-04078-y)
Supplement: Supplementary file 6 — Supplementary Data 3 [file 41467_2018_4078_MOESM6_ESM.docx]

**Supplementary Dataset 3:** Model free analysis of the ^15^N relaxation rates for wt and D76N β2m.

| Residue number | WT | | | | D76N | | | |
| --- | --- | --- | --- | --- | --- | --- | --- | --- |
|  | S^2^ ± error | | τ_SMF_ ± error (ns) | | S^2^ ± error | | τ_SMF_ ± error (ns) | |
| 1 | 0,857 | 0,004 | 21,43 | 0,58 | 0,848 | 0,002 | 30,86 | 0,48 |
| 2 | 0,874 | 0,002 | 29,63 | 0,49 |  |  |  |  |
| 3 | 0,950 | 0,002 | 20,14 | 0,76 | 0,954 | 0,001 | 21,84 | 0,57 |
| 4 | 0,901 | 0,001 | 26,57 | 0,37 | 0,909 | 0,002 | 29,30 | 0,48 |
| 5 |  |  |  |  |  |  |  |  |
| 6 | 0,939 | 0,001 | 26,01 | 0,45 | 0,954 | 0,002 | 17,00 | 0,81 |
| 7 | 0,928 | 0,001 | 17,23 | 0,27 | 0,937 | 0,001 | 13,84 | 0,31 |
| 8 | 0,962 | 0,001 | 18,96 | 0,38 | 0,964 | 0,001 | 18,23 | 0,57 |
| 9 | 0,941 | 0,001 | 23,25 | 0,22 | 0,942 | 0,002 | 26,69 | 0,71 |
| 10 | 0,929 | 0,002 | 40,33 | 0,95 | 0,921 | 0,002 | 35,91 | 1,11 |
| 11 | 0,877 | 0,009 | 31,18 | 2,14 | 0,814 | 0,015 | 40,52 | 3,15 |
| 12 | 0,904 | 0,005 | 32,65 | 1,65 | 0,831 | 0,006 | 42,36 | 1,52 |
| 13 | 0,888 | 0,003 | 30,41 | 0,70 | 0,881 | 0,003 | 37,13 | 1,07 |
| 14 |  |  |  |  |  |  |  |  |
| 15 |  |  |  |  |  |  |  |  |
| 16 |  |  |  |  |  |  |  |  |
| 17 |  |  |  |  |  |  |  |  |
| 18 |  |  |  |  |  |  |  |  |
| 19 |  |  |  |  |  |  |  |  |
| 20 |  |  |  |  |  |  |  |  |
| 21 |  |  |  |  |  |  |  |  |
| 22 | 0,912 | 0,002 | 23,13 | 0,42 | 0,901 | 0,002 | 27,26 | 0,45 |
| 23 | 0,906 | 0,002 | 20,88 | 0,39 | 0,930 | 0,001 | 35,40 | 0,54 |
| 24 | 0,937 | 0,004 | 25,53 | 1,52 | 0,956 | 0,002 | 36,50 | 1,69 |
| 25 | 0,965 | 0,001 | 31,42 | 0,74 | 0,969 | 0,001 | 34,24 | 0,85 |
| 26 | 0,973 | 0,001 | 35,39 | 0,94 | 0,971 | 0,001 | 33,67 | 1,11 |
| 27 | 0,971 | 0,001 | 22,97 | 0,79 | 0,977 | 0,001 | 19,63 | 1,03 |
| 28 | 0,970 | 0,001 | 16,90 | 0,48 | 0,969 | 0,001 | 17,72 | 0,61 |
| 29 | 0,949 | 0,004 | 23,96 | 1,83 | 0,934 | 0,004 | 30,51 | 1,86 |
| 30 | 0,933 | 0,002 | 24,59 | 0,69 | 0,915 | 0,001 | 27,55 | 0,43 |
| 31 | 0,967 | 0,002 | 31,15 | 2,27 | 0,966 | 0,002 | 44,07 | 2,52 |
| 32 |  |  |  |  |  |  |  |  |
| 33 | 0,948 | 0,002 | 24,11 | 0,85 | 0,949 | 0,001 | 25,75 | 0,75 |
| 34 | 0,927 | 0,002 | 18,76 | 0,58 | 0,932 | 0,002 | 22,47 | 0,64 |
| 35 | 0,966 | 0,001 | 15,91 | 0,35 | 0,961 | 0,001 | 21,97 | 0,32 |
| 36 | 0,959 | 0,001 | 22,43 | 0,44 | 0,964 | 0,001 | 19,77 | 0,43 |
| 37 |  |  |  |  | 0,958 | 0,001 | 20,60 | 0,31 |
| 38 | 0,966 | 0,001 | 20,02 | 0,32 | 0,956 | 0,000 | 25,26 | 0,25 |
| 39 | 0,947 | 0,001 | 25,27 | 0,52 | 0,926 | 0,001 | 30,60 | 0,44 |
| 40 | 0,932 | 0,002 | 32,65 | 0,96 | 0,935 | 0,001 | 34,24 | 0,74 |
| 41 | 0,961 | 0,002 | 22,30 | 0,98 | 0,943 | 0,001 | 26,22 | 0,63 |
| 42 | 0,926 | 0,001 | 23,51 | 0,45 | 0,938 | 0,001 | 22,16 | 0,39 |
| 43 | 0,888 | 0,003 | 33,61 | 0,85 | 0,883 | 0,003 | 37,99 | 0,97 |
| 44 | 0,921 | 0,002 | 18,57 | 0,38 | 0,908 | 0,001 | 23,62 | 0,32 |
| 45 | 0,855 | 0,002 | 18,15 | 0,24 | 0,893 | 0,001 | 22,21 | 0,17 |
| 46 | 0,937 | 0,001 | 17,19 | 0,41 | 0,851 | 0,003 | 22,85 | 0,42 |
| 47 |  |  |  |  |  |  |  |  |
| 48 |  |  |  |  |  |  |  |  |
| 49 |  |  |  |  |  |  |  |  |
| 50 |  |  |  |  |  |  |  |  |
| 51 |  |  |  |  |  |  |  |  |
| 52 |  |  |  |  |  |  |  |  |
| 53 |  |  |  |  |  |  |  |  |
| 54 | 0,819 | 0,004 | 30,71 | 0,65 |  |  |  |  |
| 55 | 0,938 | 0,001 | 30,20 | 0,70 | 0,921 | 0,003 | 36,76 | 1,36 |
| 56 | 0,949 | 0,002 | 37,07 | 1,17 | 0,940 | 0,002 | 44,06 | 1,46 |
| 57 |  |  |  |  | 0,874 | 0,005 | 41,94 | 1,65 |
| 58 |  |  |  |  |  |  |  |  |
| 59 |  |  |  |  |  |  |  |  |
| 60 |  |  |  |  |  |  |  |  |
| 61 |  |  |  |  |  |  |  |  |
| 62 |  |  |  |  |  |  |  |  |
| 63 | 0,975 | 0,001 | 35,96 | 0,98 | 0,971 | 0,001 | 40,42 | 1,78 |
| 64 | 0,958 | 0,002 | 34,50 | 1,56 | 0,954 | 0,002 | 36,76 | 1,68 |
| 65 | 0,952 | 0,002 | 27,86 | 0,89 | 0,927 | 0,001 | 24,80 | 0,39 |
| 66 | 0,952 | 0,001 | 25,57 | 0,68 | 0,946 | 0,001 | 28,72 | 0,74 |
| 67 | 0,936 | 0,001 | 35,25 | 0,56 | 0,911 | 0,001 | 47,08 | 0,64 |
| 68 | 0,880 | 0,001 | 39,85 | 0,49 | 0,885 | 0,002 | 49,07 | 0,87 |
| 69 | 0,683 | 0,013 | 32,76 | 1,31 | 0,622 | 0,011 | 31,47 | 0,89 |
| 70 | 0,868 | 0,006 | 26,73 | 1,31 | 0,764 | 0,016 | 48,92 | 3,45 |
| 71 | 0,852 | 0,004 | 20,78 | 0,63 | 0,774 | 0,010 | 28,16 | 1,29 |
| 72 |  |  |  |  |  |  |  |  |
| 73 | 0,642 | 0,022 | 24,75 | 1,52 | 0,335 | 0,034 | 17,98 | 0,92 |
| 74 | 0,571 | 0,014 | 21,44 | 0,69 | 0,543 | 0,028 | 33,38 | 2,04 |
| 75 | 0,557 | 0,019 | 19,81 | 0,85 | 0,398 | 0,054 | 40,71 | 3,64 |
| 76 | 0,661 | 0,007 | 17,94 | 0,39 | 0,479 | 0,117 | 44,66 | 10,14 |
| 77 | 0,875 | 0,005 | 16,74 | 0,65 | 0,860 | 0,003 | 29,58 | 0,59 |
| 78 | 0,933 | 0,002 | 39,53 | 1,23 | 0,885 | 0,004 | 25,17 | 0,73 |
| 79 | 0,957 | 0,001 | 28,54 | 0,70 | 0,948 | 0,001 | 33,70 | 0,95 |
| 80 | 0,965 | 0,001 | 29,79 | 0,71 | 0,969 | 0,001 | 34,70 | 1,10 |
| 81 | 0,973 | 0,001 | 22,29 | 0,86 | 0,973 | 0,001 | 23,47 | 0,66 |
| 82 | 0,960 | 0,001 | 23,97 | 0,68 | 0,971 | 0,001 | 19,76 | 0,70 |
| 83 | 0,951 | 0,001 | 26,73 | 0,31 |  |  |  |  |
| 84 | 0,954 | 0,001 | 24,44 | 0,79 | 0,951 | 0,001 | 30,07 | 0,51 |
| 85 |  |  |  |  |  |  |  |  |
| 86 | 0,951 | 0,003 | 36,44 | 2,29 | 0,950 | 0,002 | 38,34 | 1,92 |
| 87 | 0,955 | 0,002 | 19,26 | 0,73 | 0,936 | 0,001 | 37,05 | 0,82 |
| 88 | 0,868 | 0,002 | 31,53 | 0,59 | 0,507 | 0,038 | 40,76 | 3,32 |
| 89 | 0,773 | 0,002 | 22,96 | 0,24 | 0,764 | 0,004 | 26,70 | 0,42 |
| 90 |  |  |  |  |  |  |  |  |
| 91 | 0,957 | 0,001 | 15,02 | 0,31 | 0,921 | 0,002 | 18,00 | 0,53 |
| 92 | 0,891 | 0,001 | 20,24 | 0,26 | 0,922 | 0,001 | 20,44 | 0,24 |
| 93 | 0,951 | 0,001 | 15,08 | 0,40 | 0,929 | 0,001 | 20,16 | 0,28 |
| 94 | 0,937 | 0,002 | 17,67 | 0,49 | 0,940 | 0,001 | 19,52 | 0,30 |
| 95 | 0,861 | 0,004 | 22,65 | 0,71 | 0,869 | 0,005 | 35,94 | 1,45 |
| 96 | 0,667 | 0,048 | 57,70 | 8,69 | 0,678 | 0,024 | 56,10 | 4,18 |
| 97 |  |  |  |  |  |  |  |  |
| 98 |  |  |  |  |  |  |  |  |
| 99 |  |  |  |  |  |  |  |  |
